# The role of fungal G protein-coupled receptors in interspecies cell–cell communication

**DOI:** 10.1093/femsre/fuag024

**Published:** 2026-05-21

**Authors:** Giovanni Schiesaro, Antonio Di Pietro, Emil Damgaard Jensen

**Affiliations:** The Novo Nordisk Foundation Center for Biosustainability, Technical University of Denmark, Kgs. Lyngby, 2800, Denmark; Present Address: Department of Biotechnology and Biomedicine, Technical University of Denmark, Kgs. Lyngby, 2800, Denmark; Departamento de Genética, Campus de Excelencia Internacional Agroalimentario ceiA3, Universidad de Córdoba, 14014 Córdoba, Spain; The Novo Nordisk Foundation Center for Biosustainability, Technical University of Denmark, Kgs. Lyngby, 2800, Denmark; Present Address: Department of Biotechnology and Biomedicine, Technical University of Denmark, Kgs. Lyngby, 2800, Denmark

**Keywords:** GPCR, fungi, classification, cell–cell communication, non-self perception

## Abstract

Fungi occupy a wide range of ecological niches in which they compete or associate with other organisms through the perception of external signals and appropriate cellular responses. Fungal G protein-coupled receptors (GPCRs) are seven-transmembrane domain proteins that mediate the perception of extracellular cues. Despite their critical importance, the majority of fungal GPCRs are currently orphan receptors with unknown ligands and functions. In this review, we summarize the current knowledge on the role of fungal GPCRs in the perception of non-self signals and address persistent inconsistencies in the literature regarding fungal GPCR classification. We propose a refined system that differentiates between characterized, proposed, and uncharacterized GPCRs. Finally, we discuss how synthetic biology tools and experimental work in the native fungal organisms can elucidate the physiological role of fungal GPCRs during interspecies cell–cell communication.

## Teaser

The review covers recent advances on fungal GPCRs, including their molecular interactions and physiological roles. Illustration created with BioRender.com.

## Main

Fungi thrive in complex environments in association or competition with other organisms (Bahram and Netherway 2022). Most fungi live in soils, where they play a crucial role in supporting plant health; however, some pathogenic species pose severe risks to crops at both the pre- and post-harvest stages (Stukenbrock and Gurr 2023). In these complex environments, fungi must discriminate between self and non-self by engaging in cell–cell communication where signals are transmitted and perceived. Self-recognition in filamentous fungi has been extensively studied in the model organism *Neurospora crassa* (Fischer and Glass 2019). Remarkably, it was recently demonstrated that the same cellular dialogue is conserved and occurs even between distantly related fungi, such as *Botrytis cinerea* and *Arthrobotrys flagrans*, leading to a form of interspecies communication (Hammadeh et al. 2022). Although some of the intracellular components have been characterized, the molecular mechanisms of cell–cell communication between fungi remain poorly understood, particularly regarding the extracellular signaling molecules and receptors involved (Haj Hammadeh et al. 2022).

G protein-coupled receptors (GPCRs) are seven-transmembrane (7TM) proteins that convert extracellular stimuli into a coordinated cellular response (Zhang et al. 2024). While some fungal GPCRs were shown to function in hyphal chemotropism toward plant roots (Turrà et al. 2014), nematode trapping (Kuo et al. 2024), or mycoparasitism (Omann et al. 2012), for the vast majority of fungal GPCRs, the ligands and biological functions are unknown. To date, only a few ligands have been identified, and the intracellular signaling cascades are only partially understood. Furthermore, so far, the ⍺-pheromone receptor *Sc*.Ste2 from the model yeast *Saccharomyces cerevisiae* is the only fungal GPCR whose structure has been resolved (Velazhahan et al. 2022). In addition, several studies have employed bioinformatic analysis to expand the number of fungal GPCR classes (Zheng et al. 2010, Gruber et al. 2013, Cabrera et al. 2015), yet follow-up studies for experimental validation of the physiological role(s) of these putative receptors are still lacking.

The scope of this review is to summarize and discuss the current knowledge on the role of fungal GPCRs in interspecies cell–cell communication (Jiang et al. 2024, Heinen et al. 2025). We outline some shortcomings in the current classification of fungal GPCRs and propose a new nomenclature, which should be helpful in aligning future bioinformatic and experimental studies. We further highlight how host invasion in fungal pathogens requires multiple perception modules and coordination of different cellular signaling cascades. Finally, we propose strategies to improve understanding of the role of fungal GPCRs in non-self perception, using a combination of targeted genetic analysis and synthetic biology tools.

## Fungal GPCR classification

**Figure 1:**
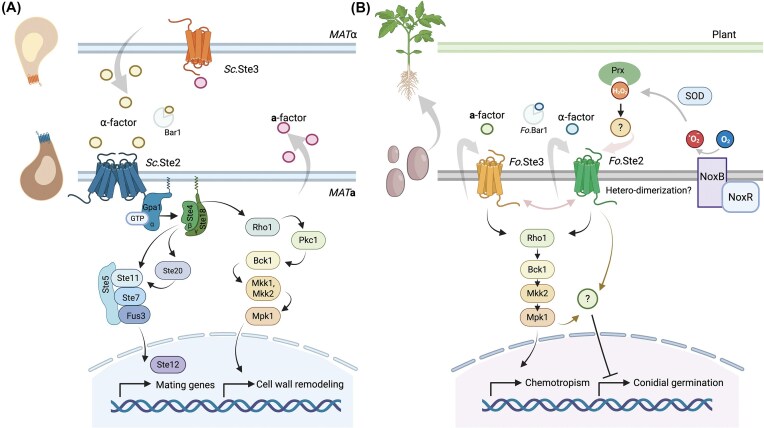
Different roles and signal transduction pathways of the pheromone receptor Ste2 in the yeast *Saccharomyces cerevisiae* (*Sc*) and the fungal phytopathogen *Fusarium oxysporum* (*Fo*). (A) Due to a conformational change upon interaction with the ⍺-factor pheromone, the GPCR *Sc*.Ste2 promotes the exchange of GDP with GTP on its G⍺ protein (Gpa1), which in turn releases the GβƔ subunits that activate the pheromone response MAPK pathway. (B) In *F. oxysporum*, the two mating receptors *Fo*.Ste2 and *Fo*.Ste3 are co-expressed and coordinate the perception of *F. oxysporum* ⍺- and **a-**pheromones, respectively (self-signals), as well as chemotropism towards plant root exudates (non-self-signals) via the cell wall integrity (CWI) MAPK cascade. Illustration created with BioRender.com.

GPCRs function in the recognition and transmission of external signals across the cellular membrane (Baldwin, 1994). GPCRs are defined by a structural motif of 7TM α-helices, connected by three extracellular and three intracellular loops (Katritch et al. 2012). Commonly, the N-terminus of a GPCR faces the extracellular environment while the C-terminus faces the intracellular space and is responsible for the interaction with the heterotrimeric G proteins. Upon ligand binding, a conformational change in the GPCR leads to the activation of an associated Gα protein and its dissociation from a bound GβƔ dimer, thereby initiating an intracellular signaling response (Latek et al. 2012) (Fig. 1). Fungal GPCRs have been classified into fourteen distinct classes based on sequence, structure, and predicted function (Gruber et al. 2013, Cabrera et al. 2015). Krishnan and colleagues previously identified three additional receptor classes (Rhodopsin, Glutamate, and Frizzled) (Krishnan et al. 2012), and a recent follow-up study by Liu et al. reported their presence predominantly in Early Diverging Fungi (Liu et al. 2025).

The current seventeen fungal GPCR classes have been defined through an iterative combination of functional characterization, comparative genomics, and phylogenetic reconstructions (Zheng et al. 2010, Gruber et al. 2013, Cabrera et al. 2015), Liu et al. 2025) and are subdivided into classical and non-classical GPCRs (Brown et al. 2018). This distinction is predominantly historical: classical fungal GPCRs were discovered first, and their mechanism of action is usually better characterized, while non-classical fungal GPCRs were discovered more recently in genome-wide surveys and comparative genomic analyses, and their functional properties remain poorly understood (Brown et al. 2018). Six out of the seventeen classes (Table 1) represent the classical fungal GPCR groups, including pheromone receptors (classes I and II), carbon sensors (class III), Nitrogen/Nutrient sensors (class IV), cAMP receptors (class V), and microbial opsins (class IX). The remaining eleven classes belong to the non-classical GPCRs, which include receptors with RGS domains (class VI), receptors similar to the rat growth hormone releasing factor (class VII), mPR-like/PAQR receptors (class VIII), Lung 7TM Superfamily (class X), GPCR89/ABA-GPCRs (class XI), Family C-like receptors (class XII), DUF300 superfamily/PsGPR11 (class XIII), Pth11 and Pth11-like (class XIV) and the three recently described classes Rhodopsin-like (class XV), Glutamate-like) (class XVI) and Frizzled-like (class XVII) (Liu et al. 2025).

**Table 1:** Current and proposed subdivision and nomenclature of fungal GPCR classes. The table is based on (Martín et al. 2019, (Liu et al. 2025) with some modifications.

GPCR class	Pfam Domain	Pfam ID	Described function or similarity	Example	Functionalized? Ligand? In yeast?	Current Classification	Proposed Classification
**I**	STE2	PF02116	Pheromone receptor	*Fo*.Ste2 (*F. oxysporum*)	Yes, Yes, Yes	Classical	Characterized
**II**	STE3	PF02076	Pheromone receptor	*Fg*.Ste3 (*F. graminearum*)	Yes, Yes, Yes	Classical	Characterized
**III**	Git3	PF11710	Carbon sensors	*Ca*.Gpr1 (*C. albicans*)	Yes, Yes, No	Classical	Characterized
**IV**	PQ-loop	PF04193	Nitrogen/Nutrient source sensors	*Af*.GprG (*A. flavus*)	Yes, Yes, No	Classical	Potential
**V**	Dicty_CAR	PF05462	cAMP receptor-like	*An*.GprH (*A. nidulans*)	Yes, Yes, No	Classical	Characterized
**VI**	RGS	PF00615	Complex sensor containing an RGS domain	*Mr*.GprK (*M. robertsii*)	Yes, No, No	Non-classical	Potential
**VII**	7tm_2	PF00002	Homologous to rat growth hormone-releasing factor receptors	*An*.GprM (*A. nidulans*)	Yes, No, No	Non-classical	Potential
**VIII**	HlyIII	PF03006	mPR-like/PAQR (homologous to yeast Izh, zinc regulators)	*Ss*.Paqr1 (*S. schenckii*)	Yes, Yes, Yes	Non-classical	Potential
**IX**	Bac_rhodopsin	PF01036	Microbial opsins	*Ff*.CarO (*F. fujikuroi*)	Yes, Yes, No	Classical	Characterized
**X**	Lung_7_TM_R	PF06814	Lung 7TM Superfamily or PTM1-like GPCR	*Nc*.Gpr-11 (*N. crassa*)	No, No, No	Non-classical	Uncharacterized
**XI**	ABA_GPCR	PF12430	GPCR89/ABA-GPCR	*Nc*.Gpr-12 (*N. crassa*)	No, No, No	Non-classical	Uncharacterized
**XII**	DUF3112	PF11309	Family C-like	*Nc*.Gpr-13 (*N. crassa*)	No, No, No	Non-classical	Uncharacterized
**XIII**	Solute_trans_a	PF03619	DUF300 superfamily/PsGPR11	*Tr*.Cps1 (*T. reesei*)	Yes, Yes, No	Non-classical	Potential
**XIV**	CFEM, fungal rhodopsin	PF05730, PF20684	Pth11 and Pth11-like	*Mo*.Pth11 (*M. oryzae*)	Yes, No, No	Non-classical	Characterized
**XV**	7tm_1	PF00001	7TM Receptor (rhodopsin family)	A0A010QRL5 (*C. fioriniae*)	No, No, No	Non-classical	Uncharacterized
**XVI**	7tm_3	PF00003	7TM Sweet-taste receptor of 3 GPCR	A0A1Y1ZHV7 (*R. globosum*)	No, No, No	Non-classical	Uncharacterized
**XVII**	Frizzled/Fz	PF01534, PF01392	Frizzled/Smoothened family	A0A397TYL1 (*G. rosea*)	No, No, No	Non-classical	Uncharacterized

In the current classification framework, there are several inconsistencies that will be addressed in the following paragraphs. Out of the seventeen proposed fungal GPCR classes (Table 1), only eleven have at least one representative receptor whose functionalization has been attempted, including classes I through IX, XIII, and XIV. For the remaining classes (X, XI, XII, XV, XVI, and XVII), extensive bioinformatic analyses have been conducted (Zheng et al. 2010, Gruber et al. 2013, Cabrera et al. 2015), but functional validation and ligand discovery (i.e. deorphanization) are currently lacking in the literature.

Importantly, at least eight of the seventeen GPCR classes contain receptors that perceive non-self signals. Here, we will predominantly focus on these eight classes, which are of particular relevance to understanding interspecies cell–cell communication, discussing both the evidence and knowledge gaps in the current literature.

## Fungal GPCR classes involved in non-self perception

### Class I and II: fungal pheromone receptors

Classes I and II contain fungal pheromone receptors, which are the best characterized fungal GPCRs. In the model yeast *Saccharomyces cerevisiae*, pheromone signaling initiates when two haploid cells, *MAT***a** and *MAT*⍺, expressing the *Sc*.Ste2 or *Sc*.Ste3 GPCRs, respectively, secrete cognate peptide pheromones to communicate with the opposite mating-type. The binding of the unmodified pheromone peptide, ⍺-factor, by *Sc*.Ste2 and of the lipidated **a**-factor by *Sc*.Ste3, leads to the same outcome inside the *MAT***a** and *MAT*⍺ cells, respectively: dissociation of the GβƔ dimer (Ste4 and Ste18) from the receptor-coupled G⍺ protein (Gpa1). Free GβƔ then initiates the mating response mitogen-activated protein kinase (MAPK) signaling pathway by recruiting Ste5, a scaffold protein for the MAPK kinase kinase (MAPKKK) Ste11, the MAPK kinase (MAPKK) Ste7, and the MAPK Fus3. The latter activates the transcriptional factor Ste12, which in turn controls the expression of hundreds of mating-related genes (Fig. 1A) (Roberts et al. 2000, Jensen et al. 2022). The complete picture of this intricate and fascinating molecular signaling mechanism has been described in great detail (Sieber et al. 2023).

It is worth noting that although the two pheromone receptors in *S. cerevisiae* share the same downstream signaling components, perception of the distinct pheromones results in different strategies for locating mating partners (Anders et al. 2021). Specifically, *MAT***a** cells that sense the diffusible α-factor pheromone secrete the α-factor protease Bar1, which reshapes the pheromone gradient (Barkai et al. 1998), thereby preventing premature mating commitment and promoting a random search behavior (Ghose et al. 2021). In contrast, *MAT*⍺ cells, which sense the hydrophobic **a**-factor pheromone, respond only in close proximity to a mating partner (Anders et al. 2021). Most studies, to date, have focused on ⍺-factor signaling in *MAT***a** cells, which occurs via the class I GPCR *Sc*.Ste2, mainly because the ⍺-factor pheromone can be easily obtained by chemical synthesis. The current focus on class I pheromone receptors entails a limitation, because class I receptors are only present in Ascomycota, while class II receptors are also found in Basidiomycota and in most of the Early Diverging Fungi (Coelho et al. 2017). In the Basidiomycota phylum, a huge diversification of sexual cycles and deviations from the two-mating-type system of *S. cerevisiae* has been reported (Coelho et al. 2017). In these organisms, a STE3-like GPCR is usually encoded next to a pheromone gene, and primary determinants of mating specificity inside the mating locus (Casselton and Olesnicky 1998). However, in several Basidiomycota, additional non-mating-type-specific STE3-like GPCRs have been observed (Niculita-Hirzel et al. 2008, Freihorst et al. 2016). Coelho et al. highlight three features for these GPCRs: 1) a lack of pheromone genes located in the proximity, 2) a longer C-terminal region, and 3) a lower level of intraspecific polymorphism (Coelho et al. 2017). The function for most of these non-mating-type-specific STE3-like GPCRs remains obscure with the exception of *Cn*.Cpr2 in *Cryptococcus neoformans*, which was shown to be constitutively active and to govern morphogenetic transitions (Hsueh et al. 2009), suggesting that STE3-like GPCRs may exhibit functional diversity throughout the Basidiomycota phylum.

In the Ascomycete plant pathogen *Fusarium oxysporum* (Fig. 1B), which lacks a known sexual cycle, the two pheromone receptors *Fo*.Ste2 and *Fo*.Ste3 are co-expressed and were experimentally shown to regulate density-dependent spore germination through autocrine signaling (Vitale et al. 2019). Interestingly, chemosensing of ⍺- and **a**-pheromone by *Fo*.Ste2 and *Fo*.Ste3, respectively, is relayed through the functionally distinct cell wall integrity (CWI) MAPK cascade (Vitale et al. 2019). Furthermore, *Fo*.Ste2 is required for chemotropism of *F. oxysporum* hyphae toward plant root exudates by sensing an unknown ligand that is generated upon exposure to plant peroxidases (Turrà et al. 2015). A similar role of the pheromone receptors in plant exudate recognition was recently described in other fungi such as *Fusarium graminearum* (Sridhar et al. 2023)*, Verticillium dahliae* (Vangalis et al. 2023), and *Trichoderma atroviride* (Moreno-Ruiz et al. 2020), suggesting a conserved mechanism for plant recognition between these closely related filamentous fungi (Chen et al. 2023).

Intriguingly, deletion of either of the two mating receptors in *F. graminearum* (*Fg*.Ste2 and *Fg*.Ste3) abolished the chemotropic response toward plant peroxidase (Sharma et al. 2022). Furthermore, heterodimerization of *Fg*.Ste2 and *Fg*.Ste3 was observed upon co-expression in *S. cerevisiae*, using bioluminescence resonance energy transfer (BRET) and pull-down experiments (Sharma et al. 2025). Meanwhile, GPCR heterodimerization is not required for the detection of plant chemoattractants in the fungal pathogen *Colletotrichum graminicola*, a species that lost its Ste2 homolog during evolution and relies solely on *Cg*.Ste3 (Rudolph et al. 2024). Moreover, *Cg*.Ste3 was shown to recognize maize diterpenoids in *C. graminicola* oval conidia (Rudolph et al. 2024). This result suggests that fungal phytopathogens may have evolved different mechanisms for perception of plant-secreted defence molecules. Further studies are necessary to understand if these different molecules (such as peroxidases and diterpenoids) have similar effects on the fungal membrane, or if their action is specific for certain fungal GPCRs.

The molecular mechanism for peroxidase-induced chemotropism has been characterized in *F. oxysporum* (Nordzieke et al. 2019). Peroxidase-stimulated chemotropism requires the NADPH oxidase B (NoxB) complex to convert oxygen into reactive oxygen species (ROS), which are then transformed into hydrogen peroxide (H_2_O_2_) by a secreted superoxide dismutase (SOD). Deletion of NoxB or its regulatory subunit NoxR, or chemical inhibition of SOD, abolished hyphal chemotropism of *F. oxysporum* towards peroxidase gradients (Nordzieke et al. 2019). In line with this, the Nox genes, particularly NoxR, were found to be involved in the molecular dialogue of the plant growth-promoting fungus *T. atroviride* with *Arabidopsis thaliana* (Villalobos-Escobedo et al. 2020), highlighting the fundamental role of ROS in plant recognition by fungi.

The unexpected range of functions described for fungal mating receptors in the recognition of both self and non-self-derived signals indicates that these GPCRs could also be important for fungus-fungus interspecies communication. In line with this idea, a recent pre-print article in *C. graminicola* suggests that pH perception via *Cg*.Ste3 has a key role in fungal interspecies communication (Rudolph et al. 2025).

### Class III: carbon receptors

**Figure 2:**
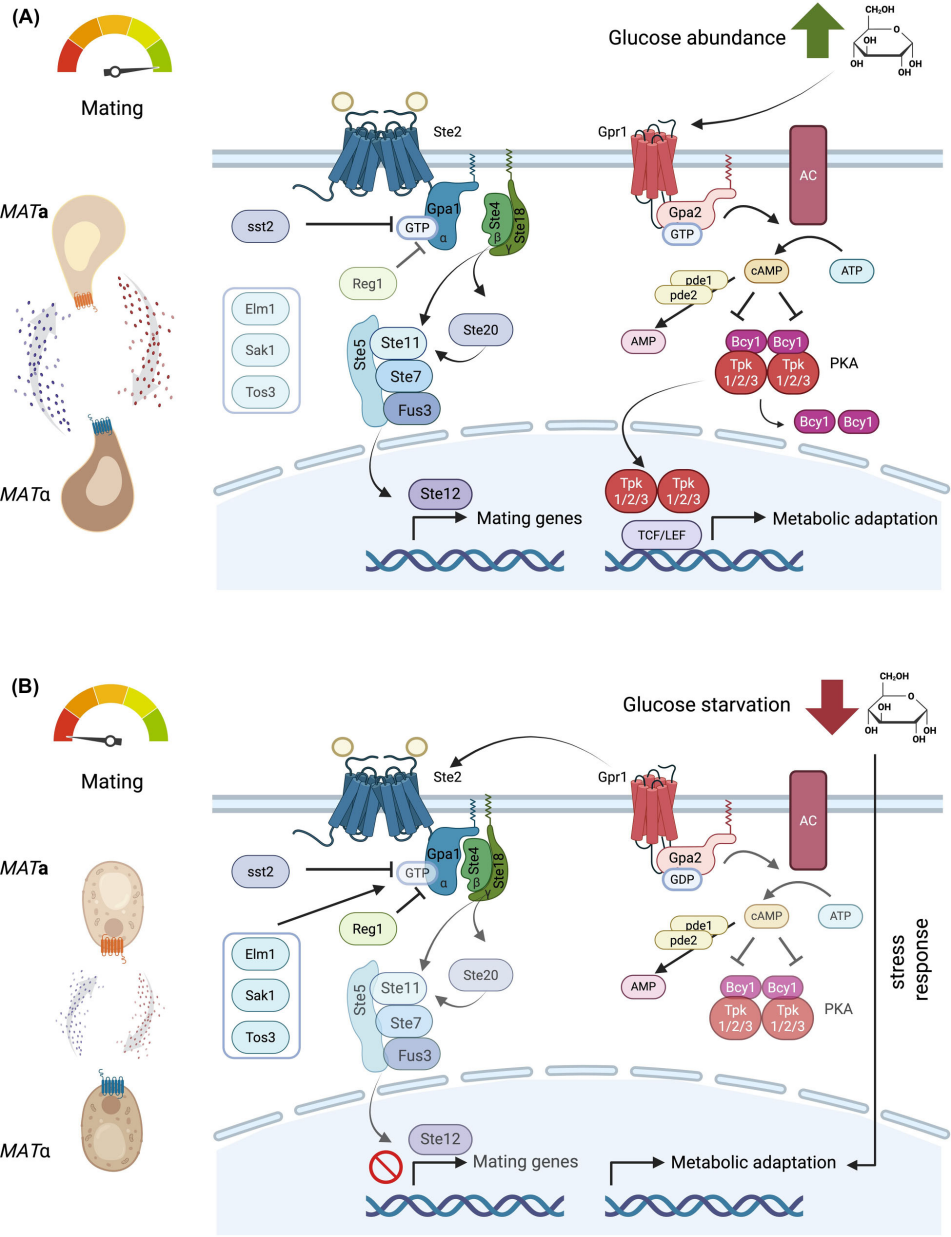
The glucose receptor Gpr1 and multiple effectors associated with the perception of glucose, control the mating response in *S. cerevisiae*. (A) Different components of the pheromone response (right) and the glucose sensing (left) pathways in *Saccharomyces cerevisiae*. (B) At low glucose levels, a metabolic response attenuates the activation of the MAPK pheromone response pathway. Illustration created with BioRender.com.

Besides the two pheromone GPCRs, *S. cerevisiae* encodes a third GPCR for sensing glucose, *Sc*.Gpr1. Upon glucose binding, *Sc*.Gpr1 activation releases a different G⍺ protein, Gpa2, resulting in downstream activation of adenylate cyclase, which catalyzes the conversion of ATP to cAMP, which in turn induces the activation of the master regulator protein kinase A (PKA) (Xue et al. 1998) (Fig. 2A). To date, no GβƔ has been found to interact with Gpa2. Despite the fact that pheromone response and glucose sensing are mediated by two distinct pathways (Versele et al. 2001), there is evidence suggesting that these two GPCR-mediated signaling cascades can interact. Glucose is not only a major regulator of yeast metabolism, but also plays a crucial role during mating (Rolland et al. 2002, Gancedo 2008, Kayikci and Nielsen 2015). In conditions of low glucose availability, Gpa1 is phosphorylated, leading to attenuation of the mating response (Clement et al. 2013). Gpa1 phosphorylation levels are regulated by the protein kinases Elm1, Sak1, and Tos3 and the protein phosphatase Reg1, which also control the master carbon response regulator Snf1 (the homolog of the human AMPK), thereby creating a direct link between glucose perception and mating response (Clement et al. 2013). In line with this, yeast mating is influenced by *Sc*.Gpr1 via control of the *Sc*.Ste2 expression level (Willhite et al. 2014) (Fig. 2B).

In *N. crassa*, the primary model organism for studying genetics, development and molecular biology in filamentous fungi, three G⍺ subunits, GNA-1, GNA-2, and GNA-3, have been identified (Turner and Borkovich 1993). In contrast to *S. cerevisiae Sc*.Gpr1, the carbon sensor *Nc*.Gpr-4 was shown to couple with GNA-1, instead of GNA-3, the homolog of Gpa2 in *S. cerevisiae* (Li and Borkovich 2006). This result highlights a difference in wiring of the carbon-sensing GPCRs and the G⍺ subunit between *S. cerevisiae* and *N. crassa* (Li and Borkovich 2006).

Class III GPCRs are not exclusively limited to carbon sensing. One of the most well-characterized class III receptors is *Ca*.Gpr1 from the opportunistic human pathogen *Candida albicans*, which controls morphogenesis and invasive growth (Miwa et al. 2004). *Ca*.Gpr1 has been reported to bind methionine and lactate, but only lactate promoted *C. albicans* survival through a potential host immunity evasion mechanism involving β-glucan masking (Ballou et al. 2016).

**Figure 3:**
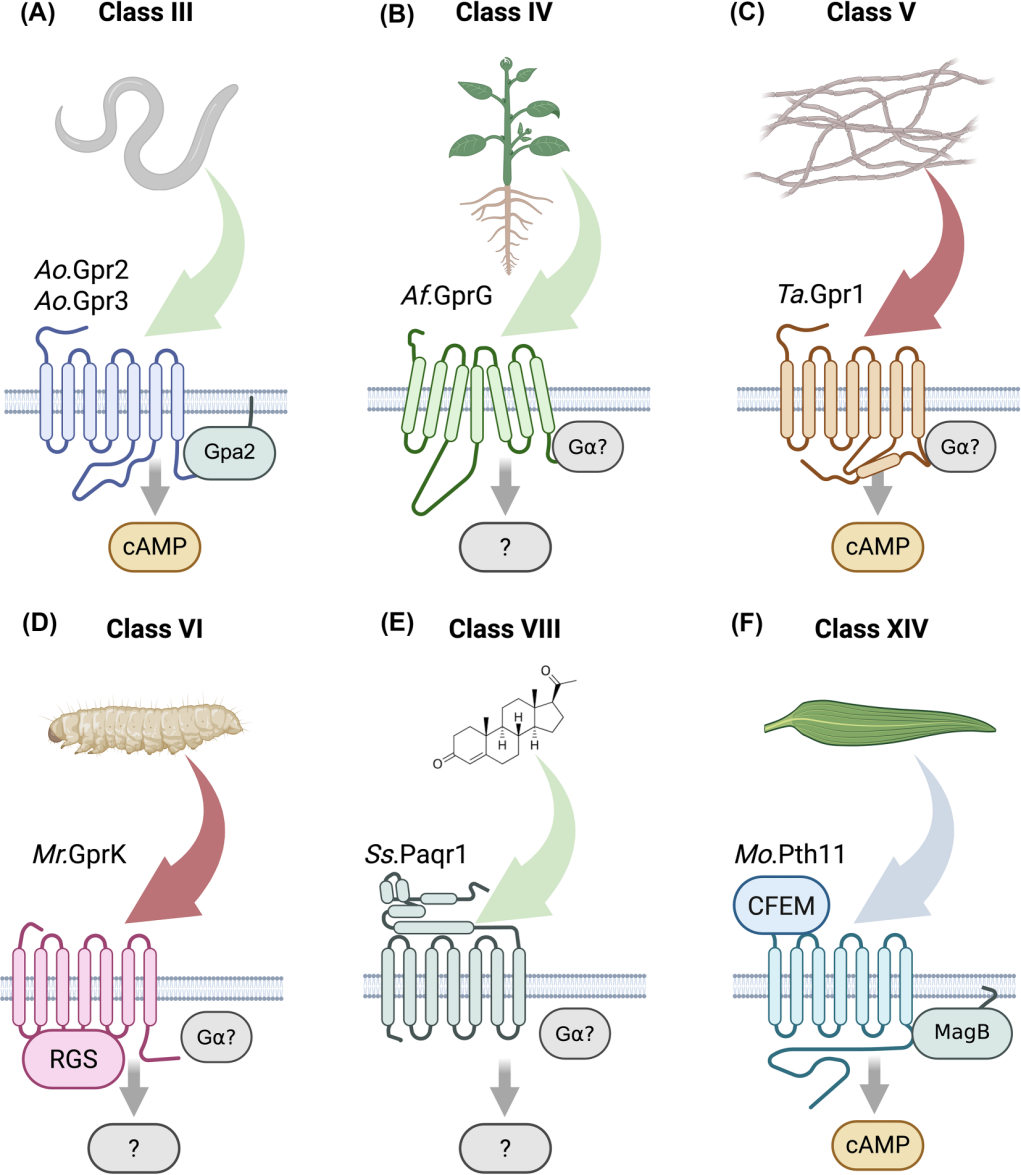
Schematic representation showing perception of different non-self signals by fungal GPCRs. One representative example per receptor class was selected. (A) Nematode pheromones (ascarosides) (Kuo et al. 2024). (B) Plant oxylipins (Niu et al. 2020), (C) fungal host (Omann et al. 2012), (D) insect host (Yu et al. 2021), (E) progesterone (Gonzalez-Velazquez et al. 2012), (F) surface hydrophobicity (Kou et al. 2017). Note that the downstream signaling cascades have only been elucidated for GPCR classes III, V, and XIV, while for the remaining classes (IV, VI, VIII), there is no evidence for association with a G⍺ protein, or the signaling cascade has not been fully elucidated. Green arrows indicate an identified GPCR-ligand association; red arrows indicate that the ligand recognized by the fungal GPCR is unknown. The blue arrow indicates that *Mo*.Pth11 senses hydrophobic surfaces, although the underlying mechanism and ligand remain unknown. Illustration created with BioRender.com.

In the nematode-trapping fungus *Arthobotrys oligospora*, three GPCRs homologous to *Sc*.Gpr1 and *Ca*.Gpr1 were identified: *Ao*.Gpr1, *Ao*.Gpr2 and *Ao*.Gpr3. *Ao*.Gpr2 and *Ao*.Gpr3 are required for trap formation in response to ascarosides (nematode pheromones), which signal via the cAMP-PKA pathway (Kuo et al. 2024). Moreover, *Ao*.Gpr2 and *Ao*.Gpr3 were shown to undergo heterodimerization in the absence of ascarosides when co-expressed in yeast (Kuo et al. 2024) (Fig. 3A). These two *A. oligospora* receptors exhibit striking structural similarity, albeit limited sequence similarity, with the nematode ascaroside receptor SRBC-66, indicative of convergent evolution (Kuo et al. 2024). Similarly, the *A. flagrans* receptor *Af*.GprC displays structural similarity to nematode GPCRs and adopts a compatible ascaroside binding pose (Hu et al. 2024). Notably, *Af*.GprC, coupled with the G⍺ subunit *Af*.GasA, has a dual localization on the membrane and on mitochondria, to boost respiration in a similar fashion to some human receptors (Hu et al. 2014, Fasciani et al. 2024).

### Class IV: nitrogen/nutrient source sensors?

Historically, class IV was defined as a group of 7TM proteins that share homology with *Schizosaccharomyces pombe* Stm1 (Chung et al. 2002). Stm1 was initially described as a nutrient-sensing GPCR (Chung et al. 2003), however more recent evidence suggests that it functions as an amino acid transporter that is localized in the vacuole and exhibits some structural similarity with GPCRs (Kawano-Kawada et al. 2021). Although it cannot be excluded that some members of this class might interact with G⍺ proteins and therefore function as canonical GPCRs (Chung et al. 2003), the limited evidence in the literature opens the possibility that class IV comprises transporters and/or non-canonical GPCRs.

Certain members of this class could potentially be involved in the perception of non-self signals. In the saprophyte *Aspergillus flavus*, four GPCRs belonging to class IV have been identified: *Af*.GprF, *Af*.GprG, *Af*.GprJ, and *Af*.GprS. Deletion of these receptors did not lead to significant phenotypic changes on different nitrogen sources tested, although *Af*.GprG has been suggested to mediate perception of the plant oxylipin 13-hydroperoxyoctadecadienoic acid (13-HpODE) (Affeldt et al. 2014). Indeed, the Δ*gprG* mutant displayed higher baseline branching, but in contrast to the wild-type strain, did not further increase branching when exposed to the fungal oxylipin 5,8-diHODE (Niu et al. 2020). Both 5,8-diHODE and 13-HpODE oxylipins have also been associated with the class III carbon sensor *Af*.GprC, although no interaction between these oxylipin sensors and G⍺ has been demonstrated (Fig. 3B).

A distinctive feature of class IV GPCRs is the presence of PQ loops in two of the transmembrane regions, which may facilitate interaction with other membrane proteins (Arines et al. 2021). However, the presence of PQ loops is not exclusive to this class of fungal GPCRs. A similar structural feature has been reported in *S. cerevisiae* Ypq2, a 7TM protein that has been implicated in the transport of arginine and histidine across the vacuolar membrane (Kawano-Kawada et al. 2019). Similarly, in *N. crassa* the two 7TM proteins *Nc*.Gpr5 and *Nc*.Gpr6, which were classified as GPCRs (Cabrera et al. 2015) were suggested to be involved in vacuolar transport of arginine and ornithine (Quinn et al. 2024).

### Class V: cAMP receptor-like

An early attempt to classify fungal GPCRs identified five classes, with class V comprising receptors that share limited sequence identity with the *Dictyostelium discoideum* cAMP receptor cAR1 (Han et al. 2004). Some of the most extensively characterized class V receptors share functional similarity with receptors from classes III and IV. These include, for instance, *Aspergillus nidulans An*.GprH, which is involved in glucose and tryptophan sensing and activates the cAMP-PKA signaling pathway (Brown et al. 2015). Similarly the *C. neoformans* GPCR *Cn*.Gpr4 was described to sense methionine, and its deletion caused defects in formation of the capsule, a known virulent factor, as well as in mating (Xue et al. 2006). In *N. crassa* mutants lacking the class V GPCRs *Nc*.Gpr1, *Nc*.Gpr2 or *Nc*.Gpr3 exhibited defects in the formation and phototropism of perithecia during sexual development (Krystofova and Borkovich 2006, Cabrera et al. 2015).

A peculiar function in non-self recognition was reported for the class V GPCR. *Ta*.Gpr1 from *T. atroviride*, which was found to be involved in fungal prey recognition during mycoparasitism against *Rhizoctonia solani* (Omann et al. 2012). Deletion of *Ta*.Gpr1 resulted in lack of host attachment and loss of mycoparasitism. Interestingly, host attachment could be restored by cAMP supplementation, suggesting that *Ta*.Gpr1 acts upstream of the cAMP-PKA pathway (Omann et al. 2012) (Fig. 3C). However, more than a decade after this report, the ligands responsible for activation of *Ta*.Gpr1 still remain unknown, and no interaction between *Ta*.Gpr1 and any of the three G⍺ subunits of *T. atroviride* (Tga1, Tga2, and Tga3) has been reported (Omann et al. 2012).

### Class VI—receptors containing an RGS domain

A defining feature of this class of GPCRs is the presence of a “Regulator of G protein Signaling” (RGS) sequence following the 7TM domain. RGS proteins serve as critical negative regulators of GPCR signaling, which act by promoting GTP hydrolysis by G⍺ proteins and signal termination (Stewart and Fisher 2015). This regulatory function enables a precise temporal control over the physiological processes governed by GPCR signaling (Soundararajan et al. 2008).

Two class VI GPCRs from *A. flavus, Af*.GprK and *Af*.GprR, have been reported to play a role in cell wall stress and oxylipin sensing (Affeldt et al. 2014). In the insect pathogen *Metarhizium robertsii, Mr*.GprK was shown to be involved in different physiological functions and virulence traits, such as appressorium formation and cuticle penetration (Yu et al. 2021) (Fig. 3D). Deletion of *Mr*.GprK led to increased intracellular cAMP levels and reduced virulence (Yu et al. 2021).

Despite the diverse functional roles reported for these GPCRs, it remains unclear whether the observed phenotypes are the result of complete loss of receptor function (sensor + regulatory domain) or are predominantly caused by loss of the regulatory RGS domain. In support of the latter, in *Aspergillus oryzae* three GPCRs with a similar architecture were found: *Ao*.GprK-1, *Ao*.GprK-2, and *Ao*.GprR. Double or triple deletions of the receptors increased production of survival structures known as sclerotia, while sclerotia formation could be restored to wild-type levels by only expressing the RGS domain without the 7TM domain (Kim et al. 2024).

Similar to other GPCR classes, the lack of knowledge of the ligands interacting with class VI GPCRs has so far limited our understanding of their physiological roles.

### Class VIII—fungal mPR-like/progesterone-adiponectin receptor (PAQR) GPCRs

The PAQR family comprises 7TM proteins with a distinctive receptor orientation, with the N-terminal domain located intracellularly (Pilon and Ruiz 2023). In human cells, the membrane progesterone receptors (mPRs) have been shown to transduce the signal through G⍺ proteins (Thomas et al. 2007). However, human mPRs have not been categorized within the GPCR superfamily, but within the progestin and adipoQ receptor (PAQR) family that has a different phylogenetic origin (Tang et al. 2005, Thomas et al. 2007).

Members of the PAQR family have been identified in multiple fungi, where they have been described as GPCRs (Brunner et al. 2008, Gruber et al. 2013). In contrast with this, in *S. cerevisiae*, four structural adiponectin receptor homologues were identified: Izh1, Izh2, Izh3, and Izh4 (Mattiazzi Ušaj et al. 2015) without being described as GPCRs. The best characterized protein, Izh2, was shown to mediate sensing of plant osmotin and induce cell death without the need for direct interaction with a G⍺ protein (Narasimhan et al. 2005).

To date, there is only a single study reporting functional analysis of *Ss*.Paqr1, a class VIII receptor in a filamentous fungus, *Sporothrix schenckii*, a soilborne organism that can cause severe skin infections (Gonzalez-Velazquez et al. 2012). By performing heterologous expression and immunoprecipitation in *S. cerevisiae*, the authors found that the *Ss*.Paqr1 interacts with the *S. schenckii* Gɑ subunit protein Ssg2 (Gonzalez-Velazquez et al. 2012). Furthermore, heterologous expression of the *Ss*.Paqr1 receptor in a model yeast-based assay (Garitaonandia et al. 2009), indicated that *Ss*.Paqr1 could mediate a response to progesterone even without co-expression of *S. schenckii* Gɑ protein (Gonzalez-Velazquez et al. 2012) (Fig. 3E). A limitation of this study is the lack of a control in which an alternative member of the PAQR family, or the native yeast Izh2 protein, was overexpressed, to prove that the observed effect on the reporter gene is indeed specific for *Ss*.Paqr1 (Gonzalez-Velazquez et al. 2012). Overall, the physiological role of progesterone sensing in *S. schenckii* and other fungi remains unclear.

### Class XIV: Pth11 and Pth11-like receptors

An interesting observation is that phytopathogenic ascomycetes have undergone a significant GPCR expansion, particularly within filamentous species of the Pezizomycotina subphylum (Xu et al. 2017). For example, the wheat pathogen *F. graminearum* has 105 GPCRs, compared to only 3 in *S. cerevisiae* or 43 in the saprophyte *N. crassa* (Cabrera et al. 2015, Jiang et al. 2019). This GPCR expansion is particularly striking for the class XIV and, although non-pathogenic fungi also have Pth11-like receptors, the expansion is far more prominent in phytopathogenic species (Brown et al. 2018).

The founding member of this GPCR class, *Mo*.Pth11, was identified in a genetic screen for non-pathogenic mutants conducted in the rice blast fungus *Magnaporthe oryzae* (DeZwaan et al. 1999). *Mo*.Pth11 contains a Common in Fungal Extracellular Membrane (CFEM) domain, characterized by eight conserved cysteine residues (Kulkarni et al. 2003). Deletion of the CFEM domain in *Mo*.Pth11 impairs appressoria formation and virulence (Kou et al. 2017), indicating that this domain is essential for perception of plant surface-associated signals and appressorium formation, acting via the G⍺ protein MagB and the cAMP-PKA pathway (Kou et al. 2017) (Fig. 3F). In contrast to *M. oryzae*, individual deletion mutants in the CFEM domain-containing GPCRs in *F. graminearum* showed no change (Jiang et al. 2019) or only partial reduction of virulence (Dilks et al. 2019). A more severe reduction in virulence was observed when five Pth11-like GPCRs, which were upregulated during wheat infection and named “GPCRs important for virulence” (GIVs), were deleted (Jiang et al. 2019). Three of these receptors were functionally analyzed and found to have different functions during infection. While the *giv1* mutant was affected in the formation of specialized infection cushions, *giv2* and *giv3* were important for infectious growth after plant penetration (Jiang et al. 2019). Interestingly, most Pth11-like GPCRs are encoded on the fast-evolving subgenome known to be enriched in genes involved in fungus-plant interaction (Wang et al. 2017). Together, these studies suggest that Pth11-like GPCRs with or without a CFEM domain might have partially overlapping functions and act in concert to coordinate virulence functions in *F. graminearum* (Jiang et al. 2019, Dilks et al. 2019).

*Neurospora crassa* was the first filamentous fungus where all available knockout mutants for this large class of GPCRs were analyzed. In this non-pathogenic species, several Pth11 family members were proposed to be involved in sensing and degradation of cellulose (Cabrera et al. 2015). This has been corroborated by studies in *N. crassa* and other species showing that G proteins regulate the production of cellulases (Collier et al. 2026, 2020), a class of secreted enzymes that act as virulence factors in many phytopathogens and are critical for carbon recycling in the environment (Kubicek et al. 2014).

An expansion of Pth11-like GPCR genes was also described in the nematode-trapping *A. oligospora* (Kuo et al. 2024), with 15 genes localizing in the subtelomeric regions known to harbor fast-evolving virulence-related genes (Priest et al. 2020). Pth11-like receptors, particularly the nematode-induced GPCR *Ao.Gin3*, were shown to be important for the perception of nematodes and pathogenicity in an ascaroside-independent manner (Kuo et al. 2024). In line with this, the importance of Pth11-like GPCRs was as well reported in entomopathogenic fungi (Shang et al. 2021). Although the repertoire of Pth11-like GPCRs in these organisms is smaller than in phytopathogens, generalist insect pathogens show a notable expansion relative to their more specialized counterparts (X. Hu et al. 2014). A deletion study including ten different Pth11-like GPCRs in the insect pathogen *M. robertsii* showed that deletion of *Mr*.Gpr8 led to loss of virulence during topical infection. Interestingly, no difference in virulence compared to the wild-type strain was observed when Δ*Mr*.Gpr8 was infected by direct injection, supporting the hypothesis that this receptor is involved in the perception of external surface components of the insect (Shang et al. 2021).

Importantly, the ligands interacting with the Pth11-like GPCRs of *F. graminearum, A. oligospora*, or *M. robertsii* during host infection are currently unknown. Identification of these ligands will be instrumental for their functionalization and the understanding of their precise roles during host infection.

## Fungal GPCRs with no evidence for a role in non-self perception

### Fungal GPCR classes VII, IX, and XIII lack direct evidence for a role in non-self communication

Class VII (fungal GPCRs similar to the rat growth hormone releasing factor), class IX (microbial opsins), and class XIII (DUF300 superfamily/PsGPR11) have each been investigated in at least one fungal organism, but no clear correlation with non-self perception or interspecies communication has been reported so far. However, for each of these three classes, there is preliminary evidence for a potential role in competition/virulence-related phenotypes.

Deletion of the class VII GPCRs *An*.GprM or *Afu*.GprM in *A. nidulans* or *Aspergillus fumigatus*, respectively, was reported to affect several physiological functions, including the production of antimicrobials or mycotoxin precursors, respectively (Dos Reis et al. 2019, Filho et al. 2020). Similarly, *Tr*.Gpr8 from *T. reesei* was shown to regulate the production of several secondary metabolites, predominantly during growth in darkness (Hinterdobler et al. 2020).

Class IX contains the opsin photoreceptors. The first receptor of this class identified was the green-light photoreceptor *Nc*.Nop-1 in *N. crassa*, which shares homology with archaeal opsins (Bieszke et al. 1999). Compared to the well-characterized human transducin coupled counterparts (Smith, 2023), the signaling function of fungal rhodopsins seems to be mechanistically distinct (Avelar et al. 2014, Fischer et al. 2016, Wang et al. 2018, Yu and Fischer 2019). For example, the *Ff*.CarO receptor from the plant pathogen *Fusarium fujikuroi* is a green light-driven proton pump that delays spore germination (García-Martínez et al. 2015). Interestingly, CarO exhibited increased pump activity when exposed to weak organic acids (García-Martínez et al. 2015) which are also important plant hormones. The same result was described for *Um*.Ops1 in the basidiomycete plant pathogen *Ustilago maydis* (Panzer et al. 2019). These findings suggest a possible role of opsin receptors might be important for fungus-plant interactions (Panzer et al. 2019) and contribute towards plant infection (García-Martínez et al. 2015).

Class XIII was created based on sequences orthologous to the oomycete *Phytophtora sojae* GPCR *Ps*.Gpr11 (Wang et al. 2010). However, *Ps*.Gpr11 failed to couple with a G⍺ protein in a yeast two-hybrid system (Wang et al. 2010), and the proteins grouped in class XIII share homology with the *S. cerevisiae* vacuole 7TM protein Hfl1 (YKR051W) (He et al. 2021). In *T. reesei*, two class XIII GPCRs, *Tr*.Cps1 and *Tr*.Cps2 have been identified, and their deletion led to a decrease in growth on cellulose (Stappler et al. 2017).

Similar to several previously described GPCR classes involved in non-self perception, these fungal GPCRs remain poorly understood at the molecular level. Further studies should focus on GPCR interactions with G⍺ proteins and on the identification of the downstream signaling cascades controlled by these receptors.

### Fungal GPCR classes lacking experimental data

Fungal GPCR classes X (Lung 7TM Superfamily), XI (GPCR89/ABA-GPCR), and XII (Family C-like) have been described in the literature, but remain entirely uncharacterized, as deletion of the encoding genes in *N. crassa* has shown no changes in phenotype (Cabrera et al. 2015).

Class X contains 7TM proteins with a Lung_7-TM_R domain. The putative GPCRs in this class share homology with *S. cerevisiae* Ptm1, a 7TM protein predicted to be localized in the Golgi and early endosome (Inadome et al. 2005). Class XI was first described in a global analysis of GPCRs in three *Trichoderma* species (Gruber et al. 2013) and subsequently in the model organism *N. crassa* (Cabrera et al. 2015). Similarly, for the receptors belonging to class XII, a classification was proposed, but no ligand or functional analyses have been conducted so far (Zheng et al. 2010, Gruber et al. 2013, Cabrera et al. 2015).

Finally, for the last three proposed classes proposed by Liu et al. (Liu et al. 2025), XV Rhodopsin, XVI Glutamate, and XVII Frizzled, which share distant homology with well-characterized human GPCRs, further experimental validation is needed.

## GPCRs of fungal pathogens activate different interconnected signaling cascades essential for virulence

**Figure 4:**
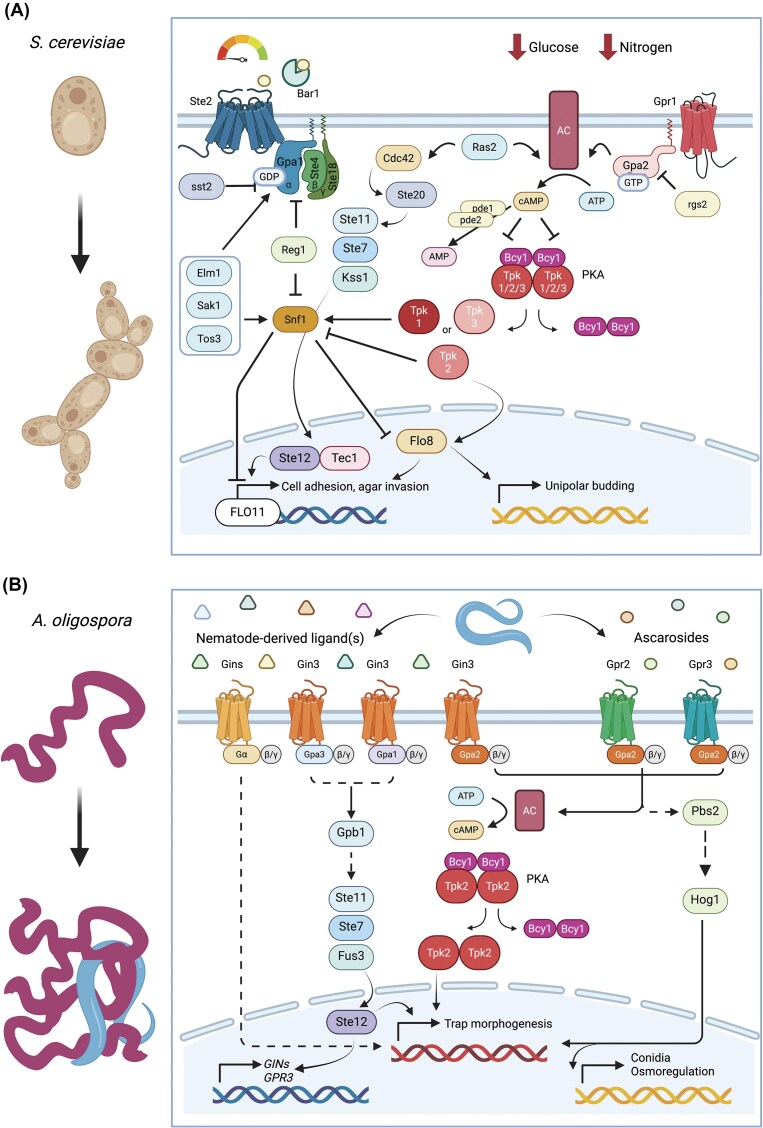
Invasive growth results from the coordinated action of different signaling cascades. (A) Nutrient starvation induces pseudohyphal differentiation and invasive growth in diploid cells of *S. cerevisiae*. This response requires multiple components of the cAMP-PKA and invasive growth MAPK pathway. (B) *A. oligospora* senses nematodes through multiple GPCRs and induces trap morphogenesis through coordinated action of the MAPK and cAMP-PKA pathways. Figure B was adapted from the proposed model by Kuo et al. (2024). Illustration created with BioRender.com.

The crosstalk between the *Sc*.Gpr1 glucose sensing GPCR and the mating cascade in yeast (see section on class III GPCRs) is not the only example of an interconnection between the MAPK cascade and the cAMP-PKA signaling pathway. Diploid cells of *S. cerevisiae* can undergo pseudohyphal growth, also known as invasive growth, in response to nutrient limitation (Roberts and Fink 1994) (Fig. 4A). This morphogenetic transition requires coordinated signaling of the MAPK pheromone response pathway, the Ras2/cAMP-PKA pathway, the AMP-activated protein kinase Snf1 and the target of the rapamycin (TOR) pathway (Kumar 2021). However, in the pheromone response pathway, neither the pheromone receptors (*Sc*.Ste2, *Sc*.Ste3) nor the upstream signaling effectors (Gpa1, Ste4, Ste18) are required for pseudohyphal growth (Cullen and Sprague 2012). Instead, the response is mediated by a series of downstream effectors, including the protein kinase module composed by Ste20, Ste11, Ste7, and the MAPK Kss1, as well as the transcription factors Ste12 and Tec1 (Cullen and Sprague 2012). In addition, pseudohyphal growth requires *Sc*.Gpr1 and its downstream cascade, which is controlled through *Sc*.Gpa2 and PKA (Lorenz et al. 2000). The functional link between MAPK and cAMP-PKA signaling pathways is provided by the small G protein Ras2 (Sengupta et al. 2007). Under glucose starvation, Ras2 is activated and interacts with the adenylate cyclase, resulting in an increase of intracellular cAMP (Toda et al. 1985). Meanwhile, Ras2 also stimulates the Cdc42p/Ste20p/Kss1 MAPK cascade mentioned above (Mösch et al. 1999). The regulation of yeast pseudohyphal growth has been described in great detail by Kumar (Kumar 2021).

Crosstalk between different signaling cascades has also been reported during host invasion by fungal pathogens. Infectious growth involves multiple signaling cascades, which are controlled by multiple GPCRs. In the nematode trapping fungus *A. oligospora*, class III GPCRs *Ao*.Gpr2 and *Ao*.Gpr3 detect host ascaroside pheromone, leading to the dissociation of *Ao*.Gpa2, which activates cAMP production (Kuo et al. 2024). Additionally, the class XIV Pth11-like receptor *Ao*.Gin3, which senses unknown nematode components, has been proposed to regulate cAMP-PKA signaling while simultaneously activating the MAPK signaling cascade (Kuo et al. 2024) which is essential for full virulence (Chen et al. 2021) (Fig. 4B).

Similarly, the Pth11-like receptor from *F. graminearum, Fg*.Giv1 has been proposed to function both upstream of the cAMP-PKA and the MAPK cascade (Jiang et al. 2019). Both pathways are known to play essential roles in virulence-related functions (Jenczmionka et al. 2003, Urban et al. 2003, Jenczmionka and Schäfer 2005, Hu et al. 2014, Bormann et al. 2014). It is therefore reasonable to infer that GPCRs acting upstream of these two conserved signaling cascades could play important roles in coordinating such complex behaviours (Dilks et al. 2019). Further studies are needed to address the molecular details of GPCR signaling in the synergy between cAMP-PKA and MAPK pathways during host infection.

It is important to note that in *S. cerevisiae*, five distinct MAPKs, Fus3, Kss1, Mpk1/Slt2, Hog1, and Smk1, regulate pheromone response, filamentation and invasive growth, cell wall integrity, high‐osmolarity stress response, and spore wall assembly, respectively (Chen and Thorner 2007), whereas most ascomycete fungi only have three MAPKs homologous to Fus3/Kss1, Mpk1, and Hog1 (Turrà et al. 2014). In *F. oxysporum*, these three MAPKs were shown to contribute in a coordinated manner to the remarkable physiological and pathogenic versatility of this cross‐kingdom fungal pathogen (Segorbe et al. 2017).

Due to their pivotal role upstream of distinct interconnected signaling cascades, fungal GPCRs represent promising targets for novel pest management drugs (Velazhahan et al. 2023). Uncovering the elusive ligands sensed by fungal GPCRs could lead to the development of targeted and specific strategies to control infection by fungal pathogens. Similar to the mating response, which is either activated or suppressed depending on the presence or absence of glucose (see Class III section), the addition of the appropriate competing ligand molecules may interfere with the pathways of host invasion in phytopathogenic fungi. Our recent study in *F. oxysporum* revealed that supplementation of natural and synthetic pheromone agonists reduces plant root penetration in the first days of infection (Schiesaro et al. 2025), highlighting the potential for targeted intervention of fungal infections through GPCR interference.

## Deorphanization of fungal GPCRs: a milestone towards developing targeted antifungal strategies

The scope of this section is to provide a general framework for the functionalization of fungal GPCRs based on 1) computational analysis, 2) employment of synthetic biology tools based on model yeast, and 3) molecular studies in the endogenous fungal systems.

### Computational approaches

Solid computational analysis can provide a strong foundation for designing relevant functionalization experiments. Compared to human cells, fungi encode fewer G⍺ proteins and usually only have a single Gβ and GƔ subunit (Xia et al. 2025). Since most annotated fungal GPCRs lack a functional link with a known G⍺ protein, an initial step for designing laboratory experiments could involve the use of AlphaFold predictions (Jumper et al. 2021, Abramson et al. 2024). Using this approach, putative interactions between novel potential GPCRs and G⍺ proteins were computationally predicted across 155 fungal genomes (Liu et al. 2025). The approach was subsequently validated experimentally in a separate study, leading to the successful identification of the G⍺ protein that interacts with the *U. maydis* GPCR *Um*.Gpe1 (Mendoza-Rojas et al. 2025).

An alternative strategy for identifying GPCRs of interest is the analysis of transcriptome data obtained during host infection. This method was used for the identification of candidate GPCRs potentially involved in host invasion by the plant and nematode pathogens *F. graminearum* (Jiang et al. 2019) and *A. oligospora* (Kuo et al. 2024). Following an analogous approach, it would be worth identifying fungal GPCRs up- or downregulated during co-culture of different fungal species.

### A yeast model for deorphanizing fungal GPCRs

Yeast offers several advantages as an experimental model, including the possibility to study individual GPCRs in a synthetic null background in which the three endogenous GPCRs have been removed (Lengger and Jensen 2020). In addition, the availability of well-established techniques for genome engineering and the robustness of yeast cells can save costs and increase the throughput of experiments (Hansson et al. 2025). Due to the similarities between the yeast mating pathway and mammalian cell signaling (Pausch 1997), yeast has been widely used as a tool to express human GPCRs (hGPCRs). Out of the ∼800 hGPCRs, almost 70 have been successfully functionalized in yeast (Deichmann et al. 2024). Because filamentous fungi are much more closely related to yeast, many of the technical difficulties encountered when employing a heterologous chassis may be mitigated, particularly those arising from the differences in membrane composition of mammalian and fungal cells, such as the presence of cholesterol versus ergosterol (Bean et al. 2022).

A general strategy for the heterologous expression of hGPCRs in model yeast has emerged over time (Lengger and Jensen 2020)(Lengger and Jensen 2020). It consists of multiple gene deletions to increase the sensitivity of the yeast biosensors by constitutive overexpression of the hGPCR and the Gα subunit (Shaw et al. 2019). Moreover, it has been demonstrated that replacing the last five amino acids of the native yeast Gα protein (Gpa1) with the sequences of human Gα proteins improves the coupling of the hGPCR with the resulting Gα chimera (Brown et al. 2000). Along the lines of this strategy, we propose that functional characterization of fungal GPCRs outside class I and II (pheromone receptors) could lead to the next leap in deorphanizing fungal GPCRs and improve understanding of their roles in fungal cell–cell communication.

**Figure 5:**
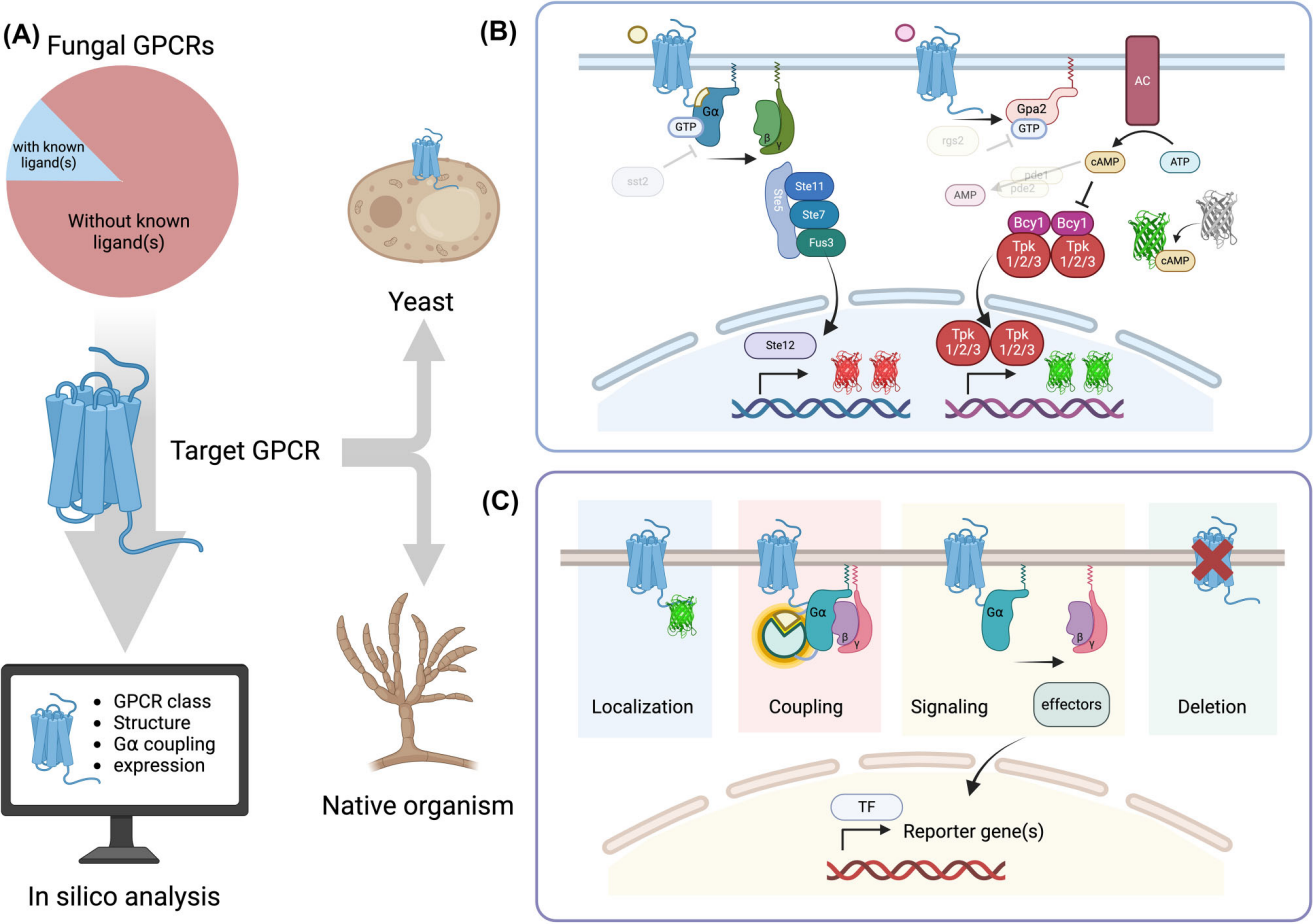
Proposed workflow for elucidating fungal GPCRs and their role in interspecies communication. (A) Computational approaches, such as modeling GPCR structure, testing coupling with G⍺ proteins, or analyzing GPCR expression profiles, will provide background knowledge and generate hypotheses that can be experimentally tested in a heterologous yeast model system (B) or in the native organism C. In the model yeast B, several genetic edits have been described to increase the sensitivity of the system and improve GPCR-G⍺ chimera coupling. A similar approach can be implemented for developing novel cAMP-PKA-based biosensors or yeast biosensors with multiple reporter genes. (C) Tools already used in human GPCR pharmacology can be implemented to study GPCRs in the native fungal system. These include luminescence and bioluminescence assays to quantify GPCR signaling, GPCR activation, GPCR-protein interaction, G protein activation, or receptor internalization. Illustration created with BioRender.com.

It is important to note that several fungal GPCRs have not shown to signal via the MAPK pheromone response cascade. For example, non-pheromone GPCRs such as those from class III and class V signal through activation of the cAMP-PKA pathway (Brown et al. 2018). Therefore, novel yeast biosensors acting as cAMP reporters (Botman et al. 2021)) as well as novel reporter genes should be designed as alternative solutions to functionalize and study these classes of GPCRs in the yeast model (Fig. 5B).

### Studying fungal GPCRs in their native systems

Although yeast is a useful platform for the deorphanization of fungal GPCRs, critical challenges remain. *S. cerevisiae* is a unicellular organism with a densely compact genome. For example, the *S. cerevisiae* genome is about five times smaller compared to that of the filamentous plant pathogen *F. oxysporum f. sp. lycopersici 4287* (12 Mb vs. 60 Mb) and contains roughly 3.2 times fewer genes (6493 vs. 21 355). While the reduced size of the *S. cerevisiae* genome provides a “cleaner” system that can be easily engineered to limit cross-talk between different signaling modules (Shaw et al. 2019), it could also lack key components required for the functional expression of certain GPCRs that evolved in specific subphyla, such as the Pth11-like GPCRs from Pezizomycotina (Xu et al. 2017). In addition, the limited knowledge on the cellular pathways involved in fungal GPCR signaling represents a potential challenge for the deorphanization of some receptor types in yeast. For all these reasons, the function of a GPCR may become apparent only within the native organism under the appropriate conditions, such as a specific stage of the fungal cell cycle (Ding et al. 2025).

An example of unsuccessful fungal GPCR-ligand functionalization was our recent attempt to couple peroxidase sensing previously demonstrated for the *Fo*.Ste2 GPCR (Turrà et al. 2015) to the yeast mating pathway response (Schiesaro et al. 2025). Nevertheless, by using yeast mating as a proxy for fungal chemotropism, we observed the robustness of *Fo*.Ste2 and its interaction with ⍺ pheromone when yeast strains expressing different Ste2 GPCRs were exposed to plant peroxidase (Schiesaro et al. 2025). This result highlights that *S. cerevisiae* can be employed for studying different aspects of GPCR-mediated fungal communication. In particular, while yeast biosensors are useful tools for ligand discovery and detection (Hansson et al. 2025), a yeast mating platform can be employed for high-throughput screening of libraries or abiotic factors, providing a good correlation with fungal chemotropism (Schiesaro et al. 2025).

Fungal GPCRs have been studied predominantly through genetic deletion in their native organisms (Cabrera et al. 2015, Jiang et al. 2019, Kuo et al. 2024). Although this can be an efficient approach for identifying the physiological role of a given GPCR, it also has important limitations such as functional redundancy or a lack of appropriate mutant phenotyping assays. Therefore, additional and complementary methods need to be considered. Tagging GPCRs with a Fluorescent Protein such as GFP could be a first step for understanding their subcellular localization and trafficking (Kallal and Benovic 2000). In addition, a set of fluorescence or bioluminescence assays have been developed in human GPCR pharmacology to quantify GPCR signaling, GPCR activation, GPCR-protein interaction, G protein activation, or receptor internalization (Pearce et al. 2025). A combination of these methods could be used to experimentally validate the interaction of fungal GPCRs with G proteins. For example, we failed to identify a single report on G⍺ interaction for any Class IV (Nitrogen sensor) GPCR in fungi. Are these receptors co-localizing with G⍺ proteins? Or is their localization restricted to the vacuole, where they could act as amino acid transporters, as shown for Stm1 in *Schizosaccharomyces pombe* (Kawano-Kawada et al. 2021)? Because such experimental methods and designs have already been developed for studying hGPCRs, it is now time to apply these principles to fungal GPCR signaling, which has historically been understudied compared to hGPCRs.

## Discussion and conclusions

Although fungal GPCRs represent promising targets for the development of novel antifungal strategies (Brown et al. 2018, Velazhahan et al. 2023), the understanding of the physiological role and the molecular mechanisms for most fungal GPCR classes is limited. There are several steps that the scientific community should take to close this knowledge gap. The current fungal GPCR classification is somewhat confusing and should be reconsidered. Here we propose a new, rather conservative, nomenclature for fungal GPCRs (Table 1), which we believe will be useful in directing future research. Instead of distinguishing between classical and non-classical GPCRs, fungal GPCR classes (I-XVII) are classified into three subdivisions, based on their degree of functional characterization:

- characterized fungal GPCRs,
- potential fungal GPCRs,
- uncharacterized fungal GPCRs.

Characterized GPCRs include the classes in which one or more members have been shown to interact with a G⍺ protein, or in which the downstream signaling cascade has been elucidated. This first subdivision currently includes the following classes:

- Class I (pheromone receptors Ste2-like)
- Class II (pheromone receptors Ste3-like)
- Class III (carbon sensors)
- Class V (cAMP receptors)
- Class IX (microbial opsins)
- Class XIV (Pth11 and Pth11-like receptors)

The subdivision “potential GPCRs” contains the fungal GPCR classes for which th ere is preliminary evidence of GPCR-G⍺ coupling or for which a downstream signaling cascade has been proposed, but not yet fully demonstrated. These include:

- Class IV (nitrogen/nutrient source sensors)
- Class VI (receptors with RGS domains)
- Class VII (receptors similar to the rat growth hormone-releasing factor)
- Class VIII (mPR-like/PAQR receptors)
- Class XIII (DUF300 superfamily/PsGPR11)

The GPCRs from the remaining classes (X, XI, XII, XV, XVI, XVII), as well as possible new future classes, are classified as uncharacterized fungal GPCRs until experimental evidence either supporting or disproving their molecular mechanisms becomes available. Such a conservative approach will raise the community’s attention to how little we know about fungal GPCRs. Only 6 out of the 17 structural classes contain at least one GPCR member that has been fully characterized in its signaling components. Therefore future studies should focus on experimental validation of G protein interactions and downstream signaling cascades of the potential and uncharacterized GPCR subdivisions. Importantly, many fungi encode receptors belonging to uncharacterized GPCR classes (Liu et al. 2025), suggesting that the role of these receptors is evolutionarily conserved across the fungal kingdom and thus must be of relevance to fungal cell physiology.

In human cells, it is well established that hGPCRs can signal either via dependent or independent G⍺ coupling (Hilger et al. 2018). Independent signaling relies on G-protein-coupled receptor kinase (GRK)-mediated phosphorylation or arrestin coupling (Hilger et al. 2018). Do fungi similarly possess similar signaling mechanisms? For example, the unexpected absence of GPCR-G⍺ interactions reported for the class V *Ta*.Gpr1 GPCR (see *Class V: cAMP receptor-like*) (Omann et al. 2012), suggests that *Ta*.Gpr1 could have an independent G⍺ protein signaling cascade.

In *A. oligospora*, 12 arrestin genes have been identified, which were differentially associated with growth, conidiation, endocytosis, pH signaling, trap formation, and pathogenicity (Zhou et al. 2022). Although virulence of *A. oligospora* is mediated by GPCRs (see above), firm evidence for a GPCR-arrestin interaction is currently lacking. Interestingly, the 7TM sensor PalH from *A. nidulans*, which is broadly conserved across ascomycetes, signals alkaline pH via the arrestin-like protein PalF (Herranz et al. 2005), thus mechanistically resembling mammalian GPCRs (Lucena-Agell et al. 2016).

Furthermore, several mammalian GPCRs can form receptor dimers or oligomer structures with other membrane proteins (Terrillon and Bouvier 2004). Gaining deeper insight into fungal GPCRs heterodimerization could advance our knowledge on the signaling functions and mechanisms of understudied receptor classes, such as those containing an RGS domain (Class VII), which might act as co-regulators of other GPCRs.

Another intriguing aspect of fungal GPCRs is the striking expansion of the Pth11-like GPCR family (class XIV) in the Pezizomycotina subphylum (see *Class XIV: Pth11 and Pth11-like receptors* section). It has been proposed that the differences in the number of Pth11-like GPCR genes between fungal species is related to genome dynamics and, in particular, with gene duplication and gene loss events (Liu et al. 2025). Although this is a reasonable hypothesis, such a trend is not observed for other fungal GPCRs classes. Because Pth11-like GPCRs have been described to mediate pathogenicity in several fungal species (Kou et al. 2017, Jiang et al. 2019, Kuo et al. 2024) this subphylum expansion could be related to increased specialization in the perception of different hosts or specific host tissues, in order to orchestrate virulence-related development during different stages of infection. Further studies should clarify if distantly related organisms have evolved functionally similar Pth11-like GPCRs to adapt to similar host or ecological niches.

Importantly, the current lack of known agonists for most fungal GPCRs severely limits their functional characterization. Uncovering the molecular nature of the signals sensed by orphan GPCRs will therefore revolutionize the understanding of fungal cell–cell communication. Furthermore, elucidating GPCR-ligand interactions will enable us to address the crosstalk between downstream signaling pathways, ultimately revealing how GPCRs convert external cues into the appropriate developmental outputs. Knowledge of GPCR agonists should also facilitate the search for antagonist molecules and potential ecological interactions between co-occurring organisms. For example, it is known that some animal venoms exert their function by antagonizing mammalian GPCRs (Van Baelen et al. 2022). A fascinating hypothesis is that communication by fungal GPCRs could likewise be modulated by natural antagonist molecules produced by competing microbial organisms.

We anticipate that the proposed approach, combining computational searches and simulations with synthetic biology and methods, already developed for hGPCRs, will greatly accelerate the advances in fungal GPCR research. Further investigations into fungal GPCR signaling through ligand interactions will advance the understanding of how fungi use self- and non-self perception to thrive across diverse ecosystems. Ultimately, these insights could lead to the development of novel GPCR-targeted antifungal strategies.
